# Comparative Study of Porcine Mesenchymal Stem Cells Behavior and Lipid Metabolism on Plant‐Based Scaffolds and Two‐Dimensional Systems for Cultivated Fat

**DOI:** 10.1002/elsc.70050

**Published:** 2025-10-13

**Authors:** Mariia Abyzova, Lasse Schoppe, Marline Kirsch, Martin Muuß, Sina Zargarchi, Jordi Morales‐Dalmau, Tuba Esatbeyoglu, Ulrich Krings, Antonina Lavrentieva

**Affiliations:** ^1^ Institute of Technical Chemistry Leibniz University of Hannover Hannover Germany; ^2^ Cultimate Foods GmbH Berlin/Hannover Germany; ^3^ Institute of Food Chemistry Leibniz University of Hannover Hannover Germany; ^4^ Department Molecular Food Chemistry and Food Development Institute of Food and One Health Leibniz University of Hannover Hannover Germany

**Keywords:** cultivated fat, cultivated meat, fatty acid profile, plant‐based scaffolds, porcine cell expansion

## Abstract

The research field of cellular agriculture has developed rapidly in recent years. Despite many successes, there is an urgent need for innovative methods to culture adherent cells. Edible scaffolds offer a promising solution for anchorage‐dependent cells from agriculturally relevant species. In this study, we present a novel approach using plant‐based scaffolds for the production of cultivated fat. Our findings indicate that coating of electrospun‐derived plant‐based scaffolds with poly‐L‐lysine significantly enhances cell adhesion and proliferation, offering a more cost‐effective alternative to coating with extracellular matrix (ECM) components. Furthermore, we investigated the influence of various adipogenic media formulations on the fatty acid composition of the cultivated fat. Notably, the incorporation of intralipid significantly changed the lipid profile, leading to an increased proportion of stearic acid with a simultaneous reduction in the proportions of oleic, linoleic, and alpha‐linolenic acid. This modulation allows for the customization of lipid profiles to satisfy diverse user requirements. However, our analysis showed that both types of matrices and the basal media formulations exerted only moderate to negligible effects on the overall fatty acid composition of the cultivated fat.

***Practical application***: In this study, we evaluated the impact of cold plasma and coating treatments on plant‐based scaffold materials to improve porcine mesenchymal stem cell adhesion and growth. Additionally, the influence of different basal media formulations and the addition of intralipid on the fatty acid composition of the cultivated fat accumulated in differentiated adipocytes were examined. Our results provide valuable insights into how these variables can be adjusted to influence the fatty acid profile of differentiated cells, to meet the requirements of customers with variable nutritional and functional needs. Discovered findings can be used for further development of sustainable alternatives within the food technology sector.

**Abbreviations**
2D: two dimensional
3D: three dimensional
α‐MEM: minimum essential medium alpha
µg: microgram
µL: microlitre
µm: micrometer
AA: ascorbic acid
BODIPY: boron‐dipyrromethene
cm: centimeter
CO_2_
: carbon dioxide
CTB: CellTiter‐Blue
DAPI: 4ʹ,6‐diamidino‐2‐phenylindole
DMEM/F12: Dulbecco's modified Eagle medium/nutrient mixture F‐12
ECM: extracellular matrix
Em: emission
ePLL: epsilon‐poly‐L‐lysine
Ex: excitation
FBS: fetal bovine serum
FGF‐2: human fibroblast growth factor 2
FID: flame ionisation detector
g: gram
GC: gas chromatography
h: hour
kDa: kilodalton
L: liter
min: minute
mL: milliliter
mm: millimeter
MSC: mesenchymal stem cells
MTBE: methyl‐tert‐butylether
MUFA: monounsaturated fatty acid
nm: nanometer
NouS: NouSerum
O_2_
: oxygen
pAD‐MSCs: porcine adipose‐derived mesenchymal stem cells
PFA: paraformaldehyde
PLL: poly‐L‐Lysine
PLGA: poly(lactic‐co‐glycolic acid)
PPARƴ: peroxisome proliferator‐activated receptor ƴ
PUFA: polyunsaturated fatty acid
RGD: tripeptide arginine‐glycine‐aspartate
RT: room temperature
s: seconds
SFA: saturated fatty acid
TrypLE: TrypLE, cell dissociation enzyme
UV‐C: ultraviolet
W: watt
w/o: without

## 1 Introduction

The world´s population is expected to grow from 8.2 billion in 2024 to a peak of 9.15 billion people by the 2050s [1]. This represents a huge societal challenge for a sustainable future, food security, and food supply [2]. Along with other cellular agriculture products, the technology of cultivated meat and cultivated fat, based on in vitro cultivation of profitable cell types of agricultural species, offers more sustainable alternatives to traditional livestock farming [3]. In addition, cellular agriculture technology enables the development of customized nutritional solutions by allowing precise control of the cellular environment and metabolic processes [4]. Customized nutritionally products can address specific dietary needs, such as those related to health conditions, age, or lifestyle preferences. Fat fulfils several important functions in the human body. In addition to serving as an energy store and providing mechanical support, it also plays an important role in hormone synthesis, signaling pathways, and nerve regeneration processes [5].

At present, producing fully cultivated meat alternatives at scale remains cost‐prohibitive. As an initial step toward a fully cultivated steak, hybrid (also known as composite or blended) products combining plant‐based and animal‐cell‐derived components are the most promising candidates to be considered [6]. The cellular component of hybrid products brings beneficial meat‐like organoleptic and nutritional properties, for example, enhancing the taste of the plant‐based product and thereby increasing customer acceptance by the addition of cultivated fat [7, 8, 9]. Thus, it is crucial to develop efficient technological approaches for the large‐scale expansion of cells from agriculturally relevant species, including adaptation for expansion in 3D geometries suitable for stirred‐tank reactors, cultivation on scaffolds to support growth, and the exploration of different materials as scaffold options to optimize production.

One of the challenges in advancing cellular agriculture products is the reliance on fetal bovine serum (FBS). This media additive, derived from unborn calves, raises ethical concerns and contradicts the objective of producing cultivated meat and fat without animal suffering [10]. Due to its limited resources, FBS keeps being more expensive. Moreover, FBSs’ composition is not defined, and their composition can vary between batches. Thus, the use of chemically defined serum substitutes is necessary for a sustainable and economic upscaling of cultivated products, such as meat and fat [11, 12, 13].

In native fat, connective tissue is responsible for the organization of the adipose tissue by producing the primary structural protein collagen and other components of the extracellular matrix (ECM), enabling its functional integrity. In traditional cell culture technology and tissue engineering, decellularized matrices, along with synthetic and natural scaffolds, are commonly used to provide 3D structural support [14]. In the emerging field of cultivated meat and cultivated fat production, edible plant‐based scaffolds hold significant potential. Edible scaffolds not only support cell growth but also enable the fabrication of structured fat, mimicking the texture and composition of native adipose tissue. Such scaffolds serve as a sustainable and biocompatible alternative for supporting cell adhesion, growth, and differentiation while integrating seamlessly into the final product without the need for removal, enhancing process efficiency and scalability [15]. Plant‐based scaffolds are manufactured using technologies like electrospinning, 3D printing, freeze‐drying, and solvent casting to create porous structures for cell growth [16, 17, 18, 19]. Recently, also decellularized plant scaffolds and soy protein flakes demonstrated promising results for cultivated meat biomanufacturing [20, 21]. The plant component of hybrid products facilitated the 3D organization of cells, scaffolding them to provide the necessary intercellular and cell–matrix interactions, while bringing the texture, binding properties, and moisture retention to the final product [22].

Although plant‐based scaffolds are promising for cultivated meat, these materials remain to be fully explored in terms of their ability to support cell adhesion, growth, and differentiation [15]. Several approaches can be used to improve cell adhesion to plant‐based materials, including physical treatments such as cold plasma and coating with adhesion‐enhancing molecules [23, 24, 25]. Cold plasma treatment is widely used to modify the surface properties of various materials, enhancing their ability to support cell adhesion. By introducing reactive species to the surface, cold plasma treatment increases surface energy, roughness, and functional groups, which improves cell adhesion and proliferation on both synthetic and natural substrates [26, 27, 28]. Another strategy for enhancing cell adhesion and proliferation on plant‐based scaffolds involves the application of coatings [25]. Specifically, plant‐derived matrices can be coated with ECM components, such as fibronectin, vitronectin, or collagen [29]. Additionally, substances recognized for their adhesive properties, such as poly‐L‐lysine, may also be used in this process [30].

The cooking properties of hybrid meat alternatives are significantly influenced by their fatty acid composition [31, 32]. Saturated fatty acids (SFA) are characterized by a higher melting point, a firmer structure, and slower melting during cooking, satisfying visible fat rendering, while mono‐ and polyunsaturated fatty acids (MUFA and PUFA) are more liquid at lower temperatures and able to enhance juiciness [33, 34, 35]. However, an SFA‐rich diet is associated with higher cardiovascular risks [36], and at the same time, MUFA and PUFA are known to have cardioprotective properties [37]. Thus, it is crucial to evaluate the influence of scaffolds and culture media formulations on the final fatty acid composition and to tailor this composition to meet the requirements for health, organoleptic properties, and cooking behavior of different final products.

In this study, we evaluated the effect of scaffold pretreatment with cold plasma and coatings on cell adhesion and growth, and different media formulations as well as the use of different plant protein‐based scaffolds on adipogenic differentiation and lipid profile of cultivated porcine fat.

## 2 Materials and Methods

### 2.1 Cell Isolation and Culture

Primary isolation of porcine adipose‐derived mesenchymal stem cells (pAD‐MSCs) was performed as recommended by Williams et al. [38] with minor modifications. Cells were isolated from adipose tissue collected from a healthy 6‐month‐old pig sourced from a private slaughterhouse located within a 1‐h travel radius of the laboratory in the vicinity of Hanover, Germany. In the biosafety cabinet, the adipose tissue was minced into approximately 1 mm^3^ pieces and then incubated with half volume of 1% collagenase I and 1% dispase II solution in HBSS (Thermo Fisher Scientific, USA). After incubation, the same volume of complete culture medium Dulbecco's Modified Eagle Medium/Nutrient Mixture F‐12 (DMEM/F12, Thermo Fisher Scientific, USA) with 10% fetal bovine serum (FBS) (Thermo Fisher Scientific, USA) and 1% penicillin/streptomycin (Thermo Fisher Scientific, USA) was added to the original volume of minced adipose tissue. The resulting mixture was centrifuged for 5 min at 300 × *g*, and the pellet was resuspended in complete culture medium, filtered through 40 µm cell strainers (Sarstedt, Germany), and seeded into the T75 flask (Sarstedt, Germany) in complete culture medium. At 80%–90% confluence, isolated cells were detached using TrypLE (Thermo Fisher Scientific, USA), counted, and cryopreserved or further cultured.

After cell revitalization, the cells were seeded into T175 flasks and cultured until reaching approximately 80% confluency. Once expanded to passage 3, cells were washed with PBS, detached with 4 mL of TrypLE, and seeded on scaffolds. Cell counting was performed using a Neubauer counting chamber to determine the appropriate volumes needed for achieving the desired cell concentrations. Subsequently, cell seeding was conducted. The cells were maintained in a humidified incubator at 37°C with 5% CO_2_. The culture medium was usually refreshed every 2–4 days, depending on the cell growth rate.

### 2.2 Adipogenic Differentiation

For the adipogenic induction, DMEM/F12 (Thermo Fisher Scientific, USA) supplemented by 50 mg/L of ascorbic acid‐2‐phosphate trisodium salt (MedChemExpress, USA) or alpha‐Minimum Essential Medium Eagle (α‐MEM, Thermo Fisher Scientific, USA), both supplemented with 1% penicillin/streptomycin solution (Thermo Fisher Scientific, USA); 10 µg/mL insulin (Merck, Germany); 2 µM rosiglitazone (Selleck Biotechnology, Germany); 1 µM dexamethasone (Thermo Fisher Scientific, USA); 0.5 mM of 3‐isobutyl‐1‐methylxanthine (Merck, Germany); and 10% FBS (Thermo Fisher Scientific, USA) were used. As a serum substitute, 1% of NouSerum (NOUBIO, USA) was used. The induction phase of 3 days was followed by the lipid accumulation phase. During the 18 days of the lipid accumulation phase, 3‐isobutyl‐1‐methylxanthine was excluded from the adipogenic induction media, and this variation of media was named lipid accumulation media. The lipid accumulation media was exchanged every 3 days.

Intralipid (Merck, Germany) is a sterile, non‐pyrogenic lipid emulsion prepared for intravenous administration as a source of calories and essential fatty acids. It consists of 20% soybean oil, 1.2% egg yolk phospholipids, 2.25% glycerin, and water for injection. Mainly, soybean oil contains linoleic, oleic, palmitic, and linolenic fatty acids. Intralipid was added to the induction media and lipid accumulation media in the concentration of 75 mg/L, compared to the control group without the addition of intralipid.

### 2.3 Scaffolds

Gamma‐irradiated electrospun‐derived plant‐based 10 cm × 10 cm “Muskel Soy‐Potato 1201RN” (GelaTex, Estonia) and “Muskel Potato 0100RN” (GelaTex, Estonia) flat scaffold sheets were used in this study as scaffold material.

### 2.4 Cold Plasma Treatment

Low‐pressure (0.1–1 bar) capacitively coupled cold plasma was employed, utilizing an eight‐electrode configuration arranged circumferentially around a cylindrical geometry. The plasma was generated using atmospheric air at a radio frequency of 13.56 MHz, with a maximum power input of 100 W (plasma system type “femto” with vacuum pump and rotary glass chamber, 13.56 MHz generator, 0–100 W; Diener electronic, Ebhausen, Germany). To investigate the influence of low‐pressure cold plasma as a surface treatment for plant protein‐based scaffolds on subsequent cell adhesion and proliferation, the scaffolds were treated according to the following parameters: power and treatment time: 30 s 50 W, 1 min 25 W, 1 min 50 W, 1 min 70 W, 5 min 25 W, and 5 min 50 W.

### 2.5 Scaffold Preparation

The scaffold materials were used as entire 100 cm^2^ scaffold sheets or were prepared by precision laser cutting into 2 cm^2^ sections. The non‐sterile laser cutting process (Emblaser 2, Darkly Labs, Australia) was carried out by the university technical workshop to ensure accuracy and uniformity of the scaffolding pieces. Importantly, the scaffolds were cut without the protective plastic film to allow immediate further processing.

After cutting, the scaffold pieces were placed in petri dishes (100 mm, Sarstedt, Germany). Sterilization was achieved by exposing the scaffold cuts to short ultraviolet (UV‐C) light for 10 min in a sterilization chamber (PURION UVC box dual, PURION, Germany). After sterilization, the scaffold sections were coated and used in the experiments.

### 2.6 Pretreatment With Poly‐L‐Lysine and Vitronectin

To prepare and treat the scaffold cuts with 150–300 kDa –α‐poly‐L‐lysine hydrobromide (300 PLL) (Merck, Germany), 3.5–4 kDa ε‐poly‐l‐lysine hydrochloride (4 εPLL) and 18 kDa ε‐poly‐l‐lysine hydrochloride (18 εPLL) (Biozol, Germany) were used. First, 1 mg of PLL was weighed and dissolved in 1 mL of cell culture‐grade water, the resulting solution was filtered through a 0.2 µm PES syringe filter (Sarstedt, Germany) for sterilization. A working solution was then prepared by diluting the stock solution in sterile cell culture‐grade water to a final concentration of 0.444 µM.

Following this, individual scaffold cuts were placed in wells of a 24‐well plate (Sarstedt, Germany). For whole scaffold sheets, the sheet was rolled onto a serological pipette under sterile conditions and then transferred into a T175 cell culture flask by unrolling and flattening it using the pipette and a tweezer.

Afterward, 160 µL of the PLL solution was carefully added to each 2 cm^2^ scaffold cut, ensuring the entire scaffold surface was covered with liquid. For the 100 cm^2^ scaffold sheets, 8 mL of the PLL solution was used. The treated scaffolds were then incubated for 30 min at 37°C, after which the excess solution was removed by aspiration. The scaffold cuts in the 24‐well plates were allowed to dry overnight in a biosafety cabinet. For the whole 100 cm^2^ scaffold sheets in T175 flasks, drying was conducted over a period of 2 days. To evaluate the importance of the drying step in the PLL‐coating procedure, the respective amount of PLL was dissolved in complete culture media and applied to untreated scaffolds directly during cell seeding.

The coating with vitronectin was conducted similarly. The recombinant vitronectin stock solution (Multus Biotechnologies, UK) was dissolved in sterile distilled water up to the final concentration of 10 µg/mL, and 160 µL of the resulting solution was carefully added to each 2 cm^2^ scaffold cut, ensuring the entire scaffold surface was covered with liquid. The treated scaffolds were then incubated for 30 min at 37°C, after which the excess solution was removed by aspiration. The scaffold cuts in the 24‐well plates were allowed to dry overnight in a biosafety cabinet.

### 2.7 Cell Seeding on Plant Protein‐Based Scaffolds

**FIGURE 1:**
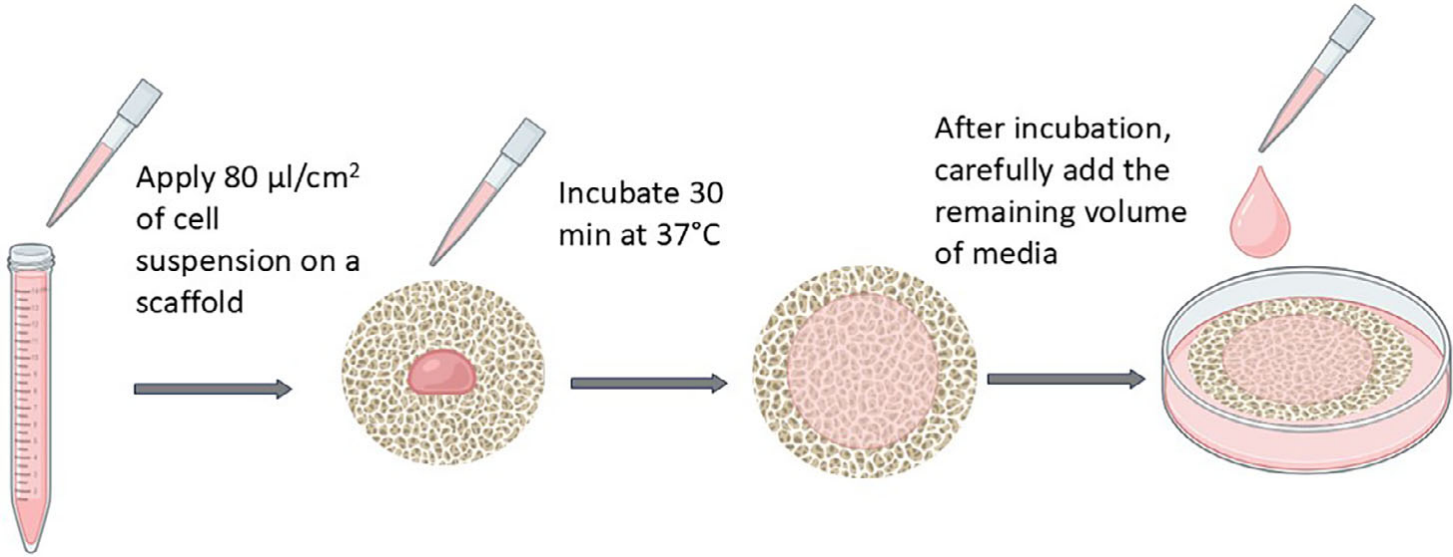
Scheme of the procedure of cell seeding on a scaffold. Created with Biorender.

The seeding procedure was conducted as shown in Figure 1. About 160 µL of cell suspension was slowly added on top of the 2 cm^2^ scaffold cuts inside the wells while trying not to break the surface tension to prevent the solution from spreading to the sides of the well. After the incubation at 37°C, 5% CO_2_ for 30 min, the remaining 860 µL complete culture medium with 2 ng/mL fibroblast growth factor 2 (bFGF2, Innocent Meat, Germany) was added, and the well plates were incubated at 39°C, 5% CO_2_ for the duration of the experiment.

### 2.8 Cell Harvesting From 2D‐ and Scaffold Culture

To harvest cells from 2D cultures, the culture medium was transferred to a 50 mL tube and centrifuged at 500 × *g* for 5 min to pellet the detached cells. During centrifugation, the remaining cell layer in the culture flask was scraped with a spatula and transferred to a cryovial (Sarstedt, Germany). After centrifugation, the pellet from the 50 mL tube was resuspended in 200 µL PBS (Thermo Fisher Scientific, USA) and added to the cryovial. The vial was then centrifuged at 300 × *g* for 5 min, the supernatant discarded, and the cells shock frozen in liquid nitrogen before storage at −80°C until further processing.

For 3D‐cultured cells, scaffolds were pooled with tweezers in 5 mL glass vials for lipid extraction or 50 mL tubes for rheological measurements. Although cells were seeded directly on scaffolds, some cells adhered to the bottom of the T175 flasks, proliferated, and differentiated in the presence of the scaffold. These cells were also harvested and analyzed separately. This remaining 2D cell layer in the flask was scraped and transferred to 5 mL glass vials. For the GC lipid profile analysis, scaffolds and cell mass were washed with sterile PBS until the supernatant was clear and centrifuged at 500 × *g* for 5–10 min between washes. After washing, the supernatant was discarded, and samples were flushed with nitrogen gas, sealed, and stored at −20°C until further use.

### 2.9 Cell Fixation and Staining

For microscopic analysis, the cells on scaffolds were fixed with a 4% paraformaldehyde (PFA) fixative solution (ROTI Histofix, Carl Roth, Germany) by aspirating the present media and adding 250 µL/cm^2^ of the fixative solution. After an incubation time of at least 20 min at RT or storage at 4°C, further staining steps were conducted.

To stain the cell nuclei with DAPI (Thermo Fisher Scientific, USA), the fixative solution was removed, and the cells were washed with 1 mL of distilled water. About 300 µL of 0.1% DAPI staining solution in distilled water was carefully added to each well and incubated for 30 min at room temperature in the dark. Subsequently, the staining solution was aspirated, samples were rinsed once with 1 mL/well of distilled water and twice with 1 mL/well of PBS (Thermo Fisher Scientific, USA).

For the lipid staining, DAPI‐stained samples were incubated with 300 µL of 0.1% BODIPY in PBS for 30 min at room temperature in the dark. After incubation, samples were rinsed two times with 1 mL per well of PBS and stored in the dark at 4°C for further analysis.

After staining, samples were analyzed with a BioTek Cytation 5 Cell Imaging Multimode Reader (Agilent, USA) using the Gen5 software (Agilent, USA).

### 2.10 Image Analysis and Quantification

Fiji software was used for the analysis of microphotographs [39]. The differentiation efficiency coefficient was calculated as the relation of the number of differentiated cells in the field of view to the total number of cells (DAPI‐stained cell nuclei), performed in per cent.

### 2.11 CellTiter‐Blue Viability Assay

The CellTiter‐Blue (CTB, Promega, USA) assay was carried out on Days 1, 4, and 8 after cell seeding to indirectly observe the viability and cell proliferation over time. For the assay, a 10% CTB solution was prepared in complete DMEM/F12 media supplemented with 2 ng/mL FGF2. The complete culture media was exchanged with 1 mL of the 10% CTB solution and incubated at 37°C, 5% CO_2_ for 2 h. The fluorometric analysis of the 100 µL sample was conducted in a 96‐well plate (Sarstedt, Germany) with the Fluoroskan Ascent plate reader (Thermofisher Scientific, USA) at an extinction wavelength of 544 nm and an emission wavelength of 590 nm.

### 2.12 Lyophilization and Total Lipid Extraction

Prior to freeze‐drying, samples were frozen for 3 h for small samples to overnight, depending on sample size. Frozen samples were lyophilized for 24 h using Alpha 1–4LSCpuls (Martin Christ Freeze‐Drying Systems, Osterode am Harz, Germany) and stored at −80°C. For the total lipid extraction, samples were treated with a modified methyl *tert‐*butyl ether /methanol (MTBE/Methanol) extraction method according to Matyash et al. [40]. Briefly, the samples were placed in pre‐weighed 5 mL glass vials and covered with an equal weight of 1:4 MTBE (Carl Roth, Germany), followed by sonication amplitude (Sonopuls, Bandelin, Germany) for 3 min with 20 s bursts and 15 s pauses at 25% of amplitude. The samples were incubated for 10 min with occasional vortexing followed by the addition of 1 mL of distilled water and additional vortexing for 20 s. To induce the three‐phase separation, the samples were centrifuged at 4700 × *g* for 10 min at 39°C. The top organic phase was transferred to a new pre‐weighed glass vial, and the extraction was repeated twice more. Finally, samples were dried under nitrogen flow for 72 h, weighed, and stored afterwards at −20°C.

### 2.13 Gas Chromatographic Analysis

For the gas chromatographic (GC) analysis, lipid extracts were prepared and fatty acids methylated according to a modified method of Christie et al. (1981) [41]. Briefly, 100 mg of each sample was dissolved in 1 mL *n‐*hexane (100 mg/mL) in a 4 mL glass vial. After gentle agitation, 1 mL of 1% methanolic sodium methoxide (CH_3_NaO) was added to allow *trans*‐esterification. The samples were heated to 60°C for 30 min, cooled to allow phase separation, and the *n‐*hexane phase was extracted twice with 1 mL of deionized water. Residual water in the *n*‐hexane phase was removed with sodium sulfate, and the dried sample was transferred to new GC vials, diluted (1:100, v/v), and stored at 4°C until analysis. The lipid profile of the cultivated fat samples was analyzed using a Shimadzu GC2010 Plus with a flame ionization detector (FID) and a DB‐5MS UI capillary column (30 m length, 0.25 mm ID, 0.25 µm non‐polar stationary phase). About 1 µL sample was injected, and the temperature program was as follows: 40°C for 5 min, then ramped to 230°C at 10°C/min, to 250°C at 5°C/min, and finally to 300°C at 10°C/min. Fatty acid methyl esters were identified by comparing retention times to a standard mixture.

### 2.14 Scanning Electron Microscopy

To evaluate the scaffold surface, scanning electron microscopy was performed. The potato protein‐based scaffold treated with 3,5‐4 kDa ε‐poly‐L‐lysine was dried at room temperature. Both treated and untreated scaffolds were covered with carbon using EM SCD500 High vacuum sputter coater (Leica Microsystems, Germany) and subsequently analyzed on JSM‐7600F Schottky Field Emission Scanning Electron Microscope with JEOL software (JEOL Ltd., Japan).

### 2.15 Statistical Analysis

Statistical analysis was performed by one‐way ANOVA tests using OriginLab 2022 software (OriginLab, USA).

## 3 Results and Discussion

### 3.1 Influence of Scaffold Treatment on Cell Adhesion and Growth

Two types of plant protein‐based scaffolds were selected for this study: potato soy protein‐based and potato protein‐based fibrous scaffolds. The choice of potato‐based scaffolds is scientifically justified by their favorable mechanical properties following hydration. Upon swelling in culture media, the potato protein‐based scaffold forms a soft, gel‐like structure, which is beneficial for promoting adipogenic differentiation in MSCs. In contrast, the potato‐soy scaffold remains a more solid, flat structure, providing a distinct mechanical environment for cell culture. Xiang et al. demonstrated that softer 3D scaffolds enhance adipogenesis compared to stiffer ones, likely due to favorable mechanotransduction pathways. Thus, using a potato protein‐based scaffold aligns with our goal of optimizing adipogenic differentiation for cultivated fat production [42].

**FIGURE 2:**
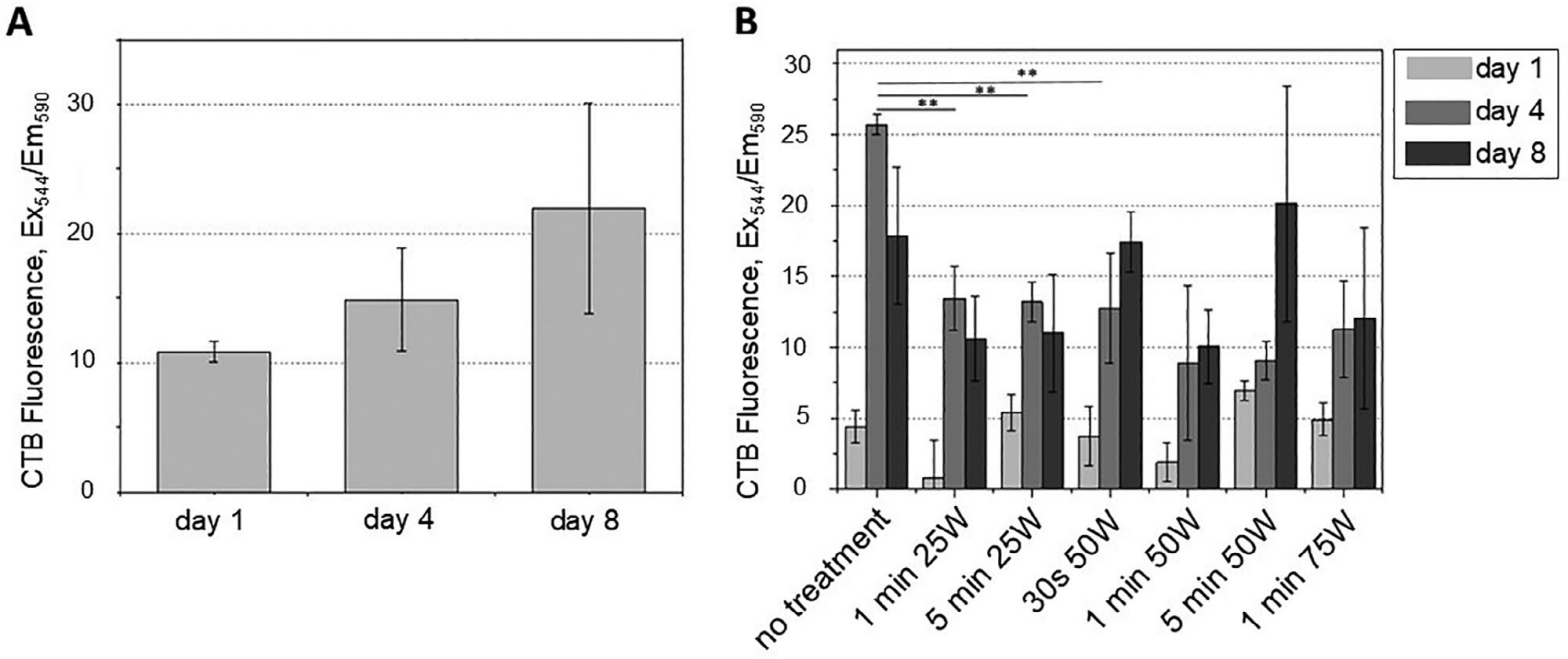
Quantification of pAD‐MSCs growth on scaffolds using CTB assay: (**A)** untreated potato protein‐based scaffolds, (**B**) cold plasma‐treated samples. **p < 0.01. One‐way ANOVA was used for statistical evaluation.

Optimizing the production of structured cultivated fat starts with maximizing cell adhesion so that the maximum amount is available on the scaffolds after seeding. This approach enables an efficient, reproducible, and robust process. To investigate cell adhesion on potato protein‐based scaffolds, first, non‐treated samples were seeded with pAD‐MSCs, and cell growth was evaluated over an 8‐day cultivation period by CellTiter‐Blue viability assay (Figure 2A).

In the course of 8 days, the number of viable cells on the untreated scaffolds nearly doubled, showing a growth rate that is insufficient for meeting the demands of potential industrial applications [43].

Despite the inherent biocompatibility of plant‐based scaffolds, recent investigations have revealed that the utilization of decellularized spinach leaves as a scaffold material results in diminished cell proliferation when compared to cell culture flasks. This can be attributed to the insufficient mechanical stiffness of the spinach leaf scaffolds, which may hinder cell adhesion and subsequent proliferation [44]. Furthermore, studies involving scaffolds composed of pea protein isolate have demonstrated suboptimal metabolic activity and proliferation rates during the expansion phase of the cells [45].

Cold plasma is widely used to treat surfaces to enhance cell adhesion, while also providing the added benefit of material sterilization [26, 27, 28]. The specific treatment parameters utilized in this study were selected based upon prior investigations, including phenolic compounds that revealed a particular sensitivity of the sample material to prolonged plasma exposure due to increasing the temperature and prolonged cold plasma reactive oxygen and nitrogen species, which could change the protein structure and affect the enzyme activity [46]. These treatment conditions were aligned with surface modifications achieved in a related study examining the impact of cold plasma on zein‐based films, thereby providing a comparative context [46]. An atmospheric cold plasma treatment was applied to potato‐based scaffolds to facilitate pAD‐MSC adhesion. Variable time exposures and power settings were applied to the scaffolds. After the cold plasma treatment, the scaffold samples were re‐sterilized and seeded with cells. Cell viability was evaluated on Days 1, 4, and 8 after seeding by CellTiter‐Blue viability assay. Atmospheric cold plasma treatment of potato‐based scaffolds with tested settings of exposure time and applied power does not improve cell adhesion and viability on scaffolds (Figure 2B). Although surface modification by plasma treatment has been shown to promote cell adhesion on various materials, including polycaprolactone [47], zein films [48], and decellularized animal‐derived matrices [49], it appears to be less effective for potato protein‐based scaffolds under the selected conditions.

During laboratory handling, cold plasma‐treated scaffolds appeared noticeably more hydrophilic than untreated scaffolds by their rapid and uniform wetting when exposed to aqueous solutions. This effect is likely due to the introduction of additional polar functional groups on the scaffold surface during cold plasma treatment in the presence of atmospheric oxygen, which enhances the material's ability to interact with water molecules [50‐52]. Future studies should incorporate quantitative assessments of hydrophilicity (e.g., via contact angle goniometry), surface charge (zeta potential measurements), and chemical composition (XPS/FTIR) to further correlate material properties with cellular responses. This would enable more rigorous optimization of scaffold surface engineering approaches. Furthermore, qualitative handling of poly‐L‐lysine as well as vitronectin‐treated scaffolds was observed, suggesting reduced electrostatic charge accumulation compared to untreated scaffolds, though surface potential measurements were not quantified. Additionally, a slight increase in hydrophilicity was noted, although these effects were not quantified through direct surface potential or contact angle measurements.

The results presented in Figure 2A were derived from the same scaffold treatment protocol as the “no treatment” sample shown in Figure 2B. However, some discrepancies are evident, with cells in the Figure 2A showing a higher CTB signal after 8 days on the same scaffold type. This variability likely reflects inherent differences in cell adhesion and proliferation among biological replicates in our experiments, which can arise under identical experimental conditions.

**FIGURE 3:**
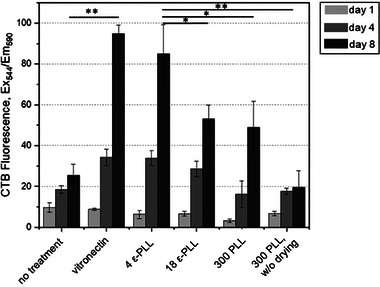
The effect of different coatings on cell adhesion and proliferation on potato protein‐based scaffolds. 150–300 kDa poly‐L‐lysine with and without drying (300 PLL), (300 PLL w/o drying), and ε‐poly‐L‐lysine (ε‐PLL) of different chain lengths: 3.5–4 kDa (4 ε‐PLL) and 18 kDa (18 ε‐PLL). Data are presented as the mean with standard deviation. *p < 0.05, **p < 0.01. One‐way ANOVA was used for statistical evaluation.

pAD‐MSCs are anchorage‐dependent cells whose metabolism and proliferation depend on adhesion to the substrate. This adhesion occurs between the cell adhesion molecules in the cell membrane and ECM proteins with specific motifs (e.g., RGD motifs). Plant‐derived scaffolds, however, lack several important bioactive sequences that are typical of the mammalian ECM and are required for effective cell adhesion. Therefore, another approach to enhance cell adhesion and proliferation is to coat the scaffolds with molecules that promote attachment, such as the commonly used vitronectin and fibronectin [53]. However, the high cost of these compounds makes them prohibitive for the production of cultivated products. Poly‐L‐lysine is a synthetic polymer made up of multiple lysine residues. It is positively charged, which allows it to interact electrostatically with negatively charged cell membranes. This makes it useful for promoting cell adhesion to various surfaces and it is more cost‐effective than integrins [54]. Due to the presence of two amino groups, lysine can polymerize in different ways depending on whether the α‐amino group or the ε‐amino group is involved in the formation of the peptide bond. In this study, vitronectin, α‐poly‐L‐lysine (PLL), and ε‐poly‐L‐lysine (εPLL) were used for the coating of the scaffolds. As expected, the highest cell adhesion and proliferation were observed on vitronectin‐treated scaffolds (Figure 3), since this ECM protein enhances cell adhesion by interacting with integrins and promoting focal adhesion [53]. Although vitronectin can be produced recombinantly, its high cost and production complexity make it unsuitable for cost‐effective industrial‐scale production of cultivated fat. Comparable results were found on scaffolds treated with PLL. In particular, low molecular weight (3.5–4 kDa) εPLL facilitates cell adhesion and proliferation to a higher extent than 18 kDa εPLL and 150–300 kDa PLL. However, the addition of PLL in culture media during the seeding procedure (300 kDa PLL without drying) did not improve cell adhesion and viability on scaffolds, emphasizing the importance of the drying step before seeding (Figure 3).

PLL is a widely used polymer for both biomedical and cell culture applications due to its versatile properties. For example, PLL has been shown to promote cell adhesion in mammalian cell culture, and it was further proven that it enhances cell growth on poly(lactic‐co‐glycolic acid) (PLGA) microspheres [55, 56]. It was also observed that PLL improved cell adhesion, migration, and overall cell viability, including enhanced proliferation in alginate microgels for cell encapsulation [57]. Lee et al. also reported that PLL has a molecular weight‐depended effect on adipogenic differentiation of 3T3‐L1 cells, probably due to interaction with insulin receptor [58]. Due to its good performance and cost‐effectiveness, 3.5–4 kDa εPLL was chosen over vitronectin as the coating reagent for subsequent experiments.

**FIGURE 4:**
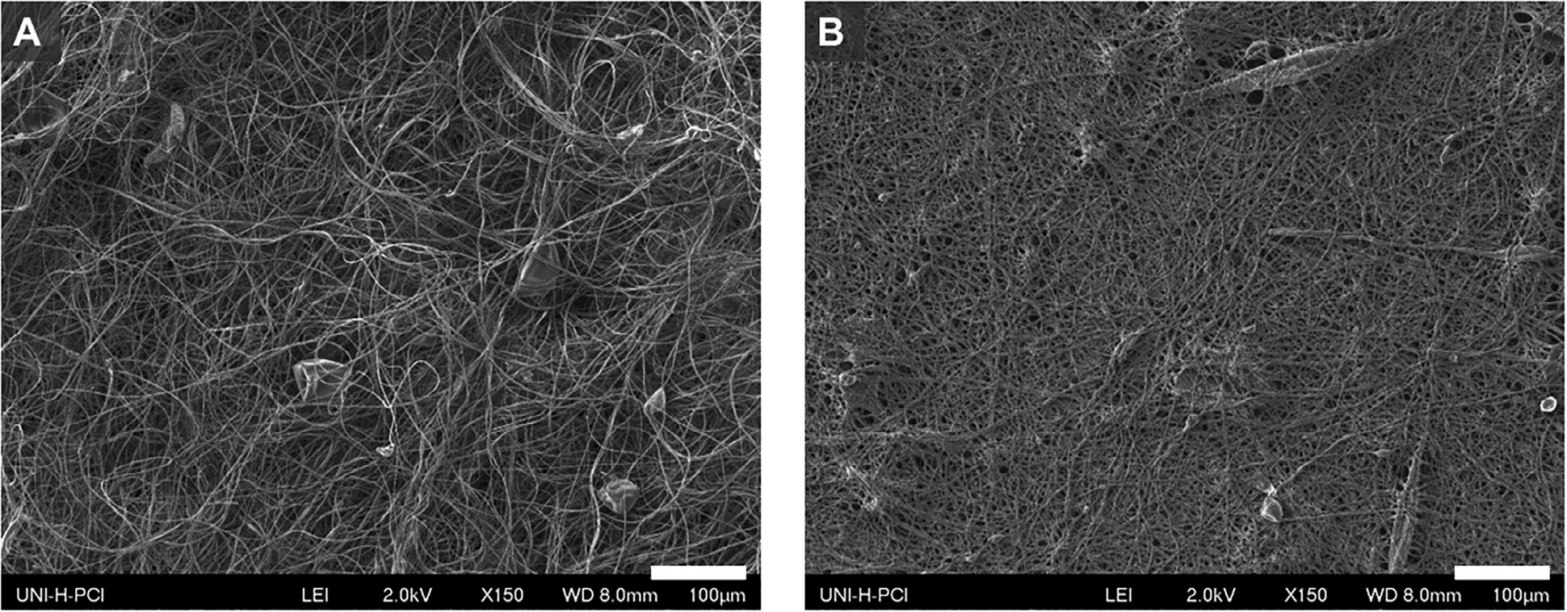
Scanning electron microphotographs of an untreated (A) and treated with 3.5–4 kDa ε‐poly‐L‐lysine (B) potato protein‐based scaffold, magnification is ×150, the scale bar is 100 µm.

As shown in Figure 4, treating the potato protein‐based scaffold with 3.5–4 kDa ε‐poly‐L‐lysine induces notable morphological changes. Scanning electron microphotographs reveal that the initially loose fiber network of the untreated scaffold becomes denser after coating, resulting in a notable reduction in pore size. These structural changes are expected to enhance cell–scaffold interactions by increasing the available surface area for cell adhesion and subsequent proliferation. Moreover, ε‐poly‐l‐lysine is a food‐grade additive possessing antimicrobial properties, making it a promising candidate for applications in the cultivated meat industry [59, 60, 61, 62].

Adipogenic differentiation of pAD‐MSC on PLL‐coated plant‐based scaffolds was investigated next. Two types of plant materials were compared as scaffolds: potato and potato‐soy. Moreover, the impact of four experimental conditions on adipogenic differentiation was evaluated: (1) α‐MEM with 10% FBS, (2) DMEM/F12 medium supplemented with ascorbic acid and 10% FBS, (3) the inclusion of intralipid supplementation in both tested media, and (4) the addition of serum compared to a serum substitute.

**FIGURE 5:**
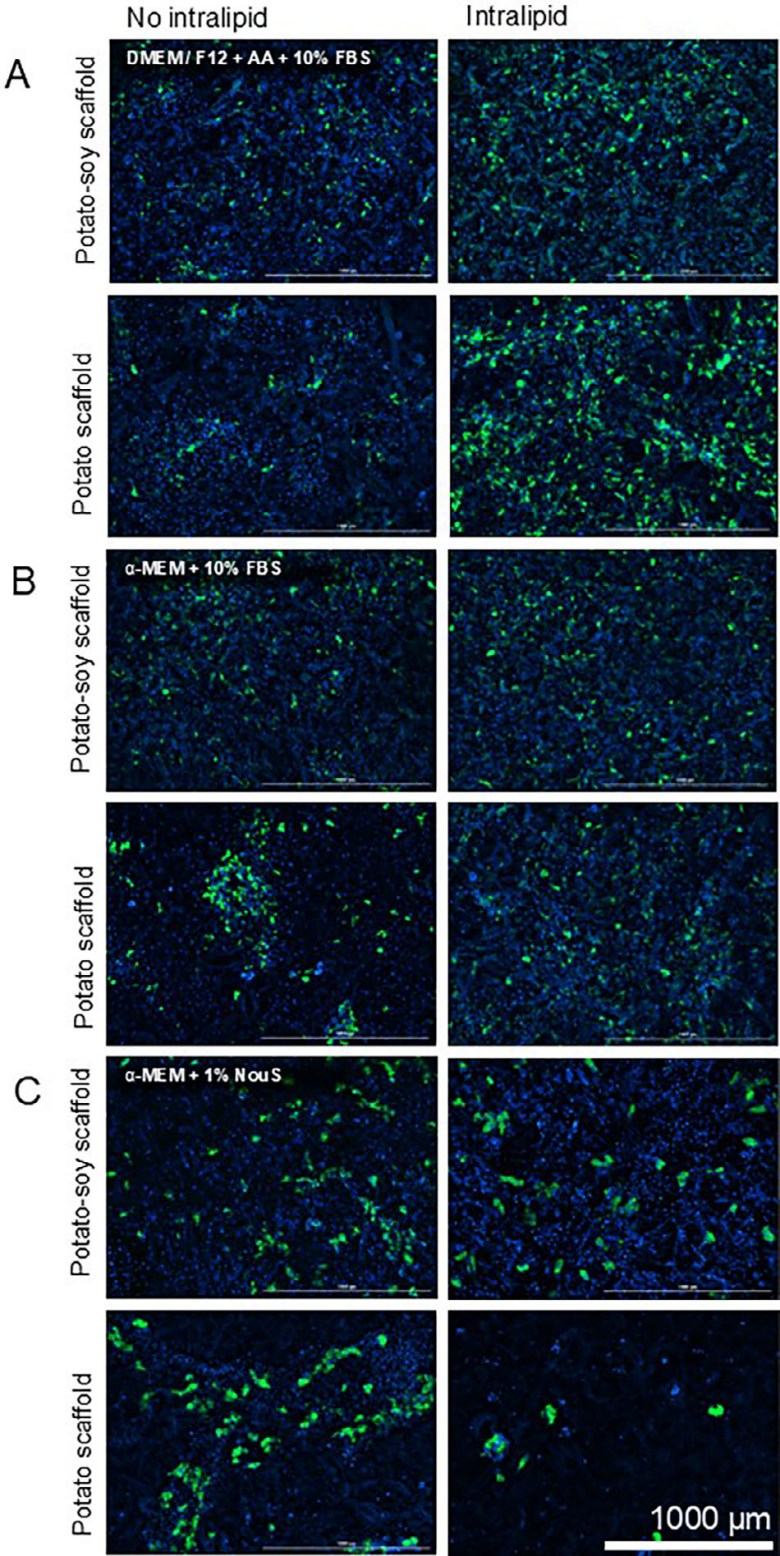
Differentiation of pAD‐MSC on plant‐based scaffolds in (A) DMEM/F12 supplemented with ascorbic acid‐based induction and lipid accumulation media with 10% FBS, (B) α‐MEM‐based induction and lipid accumulation media with 10% FBS, and (C) α‐MEM‐based induction and lipid accumulation media with 1% NouSerum. DAPI‐stained nuclei are presented in bright blue, BODIPY‐stained lipids presented in green.

Intralipid is a lipid emulsion containing soybean oil, egg yolk, phospholipid, glycerin, and water and is approved for medical use. In this experiment, intralipid was used as a source of fatty acids found in soybean oil, which can promote adipogenesis and fat accumulation in fat cells [63]. The addition of intralipid resulted in more efficient adipogenic differentiation in adipogenic media based on DMEM/F12 supplemented with ascorbic acid on both types of scaffolds (Figure 5).

Notably, cells on the potato scaffold showed better differentiation compared to those cultivated on the potato‐soy scaffold when cultured with additional Intralipid in DMEM/F12 (Figure 5A). However, this was not observed when α‐MEM was used as a basal medium (Figure 5B). When 10% FBS was replaced by 1% of the commercially available compound NouSerum (NouS), the adipogenic differentiation was comparable to serum‐containing conditions (Figure 5C). Here, the addition of intralipid did not significantly improve adipogenic differentiation. Notably, in serum‐free conditions, differentiated cells were bigger compared to cells differentiated in 10% FBS‐containing media. Furthermore, the total cell number observed on potato‐based scaffolds cultivated in α‐MEM with 1% NouS‐based induction and lipid accumulation media supplemented with intralipid is significantly lower, than that in other conditions.

For the quantitative analysis of the adipogenic differentiation of pAD‐MSC on plant‐based scaffolds, the differentiation efficiency coefficient was calculated as a relation between differentiated cells number to the total number of cells.

**FIGURE 6:**
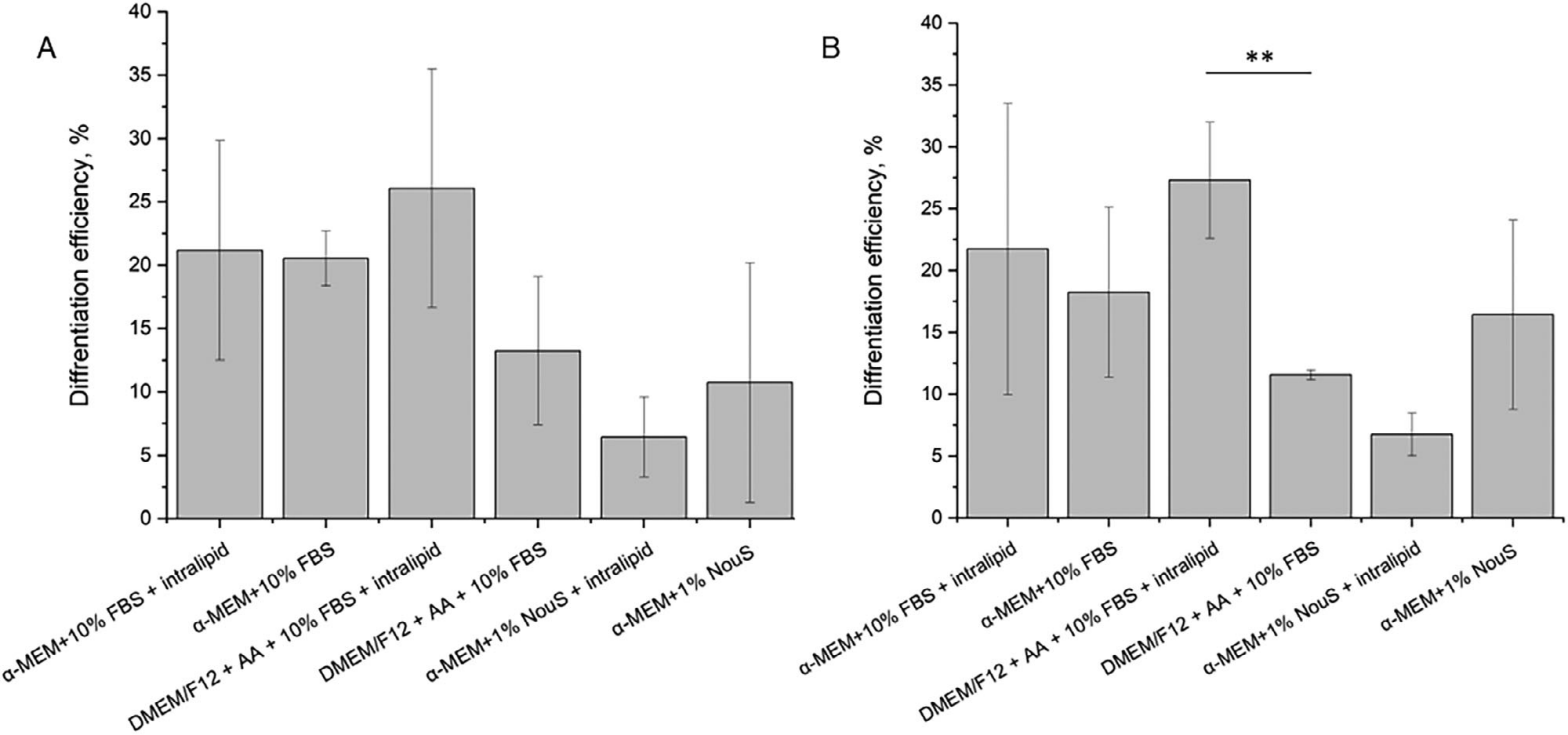
Quantitative evaluation of the adipogenic differentiation efficiency of pAD‐MSC on potato‐soy protein‐based (A) and potato protein‐based (B) scaffolds. Data are presented as the mean with standard deviation (*n* = 3). **p < 0.01. One‐way ANOVA was used for statistical evaluation.

This quantitative assessment supports the qualitative findings: on the potato protein‐based scaffold, the addition of intralipid to DMEM/F12‐based media resulted in a statistically significant increase in differentiation efficiency (Figure 6B), whereas this effect was not observed with the potato‐soy protein‐based scaffold. No statistically significant improvement was seen when intralipid was added to α‐MEM media supplemented with 10% FBS. In contrast, replacing 10% FBS with 1% NouSerum led to a non‐significant decrease in adipogenic differentiation for both scaffold types (Figure 6).

Lengi et al. demonstrated that the addition of intralipid to DMEM‐based FBS‐containing adipogenic differentiation media leads to an increase in the transcription level of peroxisome proliferator‐activated receptor gamma (PPAR‐γ), which promotes fat cell differentiation [64, 65]. As mentioned above, the key component of intralipid is emulsified soybean oil, containing linoleic, oleic, palmitic, and linolenic acids. Ruiz‐Vela et al. demonstrated that human cancer cells can be transdifferentiated into adipocyte‐like cells when cultivated with palmitoleic, oleic, petroselinic, elaidic, erucic, and linoleic acids [66]. It has also been described that cultivation of bovine stromal vascular cells with palmitic and linoleic fatty acids resulted in a dose‐dependent increase in PPAR‐γ transcript levels [67].

Lower adipogenic differentiation of cells in serum‐free media can be explained by the absence of critical growth factors, hormones, and extracellular matrix components typically provided by serum, which are necessary for the activation of adipogenic pathways and the promotion of lipid accumulation. However, for reasons of animal welfare and the development of animal‐free bioprocesses for cultured meat, the use of FBS must be avoided in the final industrial process. Our results show that although pAD‐MSCs can differentiate without FBS, both the degree of differentiation and cell numbers were lower under these conditions. This suggests that improved formulations for FBS substitutes need to be developed in the future.

To date, the impact of basal media on the adipogenic differentiation of pAD‐MSC has not been exhaustively studied. Nevertheless, DMEM is widely used for the adipogenic differentiation of pAD‐MSCs, while α‐MEM seems to be used less often for MSC [68]. In general, however, both DMEM/F12 and α‐MEM are described as the best‐performing basal media formulations for adipogenic differentiation of human adipose stem cells [69]. For this reason, we tested basal media for pAD‐MSC differentiation in this study. Despite the growing interest in plant‐based scaffolds for tissue engineering, their potential for cultivated meat and fat applications remains underexplored. However, the successful cell growth and differentiation of stem cells on these scaffolds highlight their promise for such applications [20, 70, 71].

Additionally, a quantitative evaluation of lipid accumulation was conducted. For this analysis, samples from potato scaffolds in the best‐performing media formulation (DMEM/F12 + ascorbic acid + 10% FBS) were analyzed by calculating the ratio of extracted fat mass to the dry mass of the sample. Supporting the data of fluorescent microscopy, it was shown that the yield of lipid extracted from cells cultivated with intralipid is higher than that of cells cultivated without intralipid (Table 1). Relatively low shares of fat extracted from 3D (scaffold‐based) samples can be explained by the relatively high content of scaffolding material in the resulting cultivated fat.

**TABLE 1:** Yields of extracted fat from pAD‐MSCs cultivated on 3D potato scaffolds with and without intralipid.

Cultivation conditions	Yield of extracted fat in 3D cultivated fat sample, %
Potato scaffold, DMEM/F12 + AA + 10% FBS	1.498
Potato scaffold, DMEM/F12 + AA + 10% FBS + intralipid	2.518

### 3.2 Fatty Acid Composition

The fatty acid composition of fat reflects its nutritional and physical qualities. Mono‐ and polyunsaturated fatty acids (MUFA and PUFA) are valuable components of a healthy diet due to their cardioprotective properties [72, 73, 74]. Saturated fatty acids (SFA) content characterizes fat properties important for food manufacturing and cooking. SFA demonstrate higher melting temperatures compared to MUFA and PUFA [35], which facilitates solid‐like behavior at room temperature, and their use in the food industry [75]. For the investigation of the effect of cultivation conditions (scaffold material and differentiation media formulations) on the fatty acid composition of cultivated fat, three groups of samples were generated and analyzed by gas chromatography with a flame ionization detector (GC‐FID). The first group of samples included cells cultivated in tissue culture flasks (2D); the second group was presented by cells cultivated on plant‐based scaffolds (3D), and the third group consisted of samples cultivated in tissue culture flasks (2D) in the presence of plant‐based scaffolds (but not attached to them).

**FIGURE 7:**
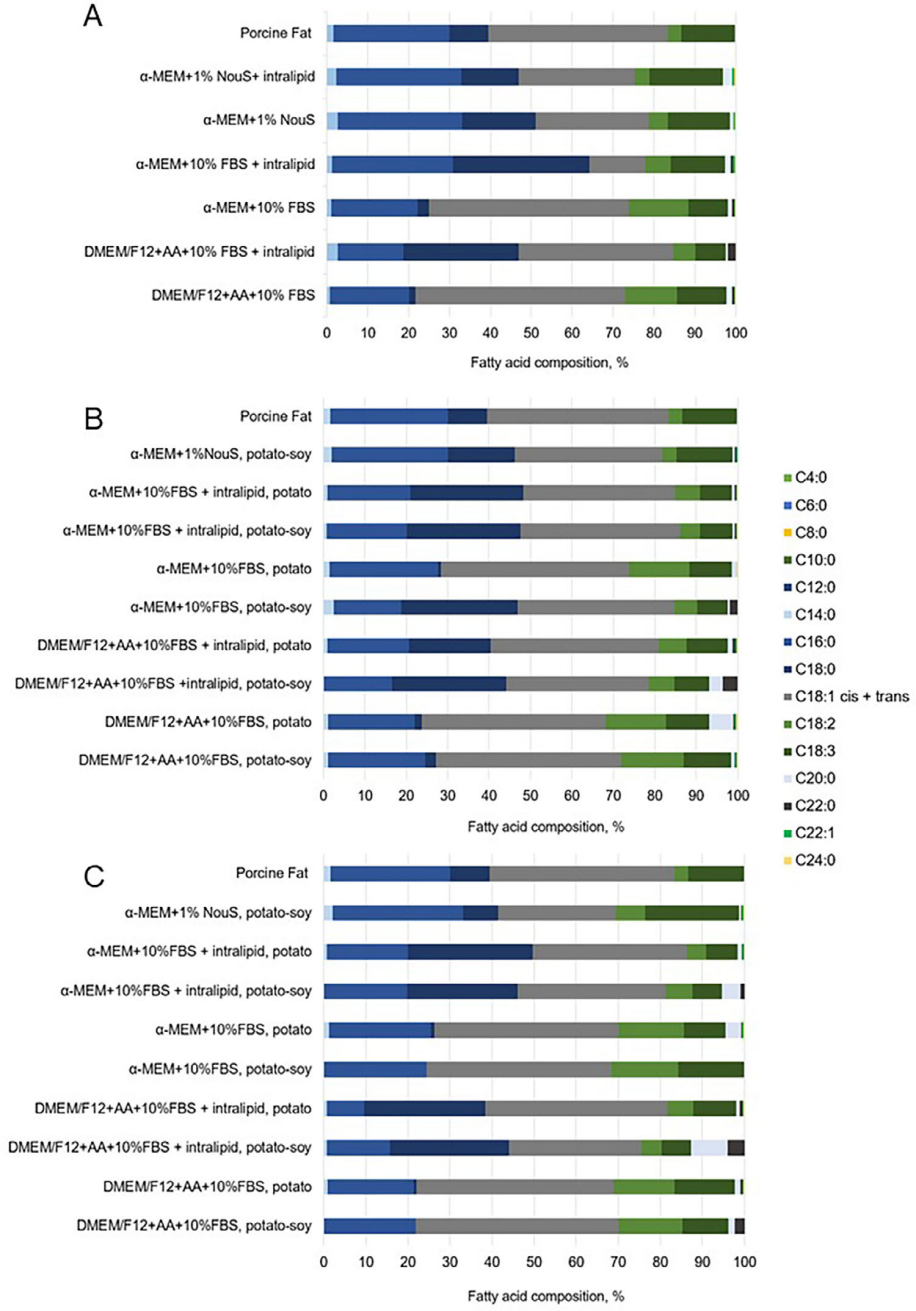
Fatty acid composition of cultivated fat measured by gas chromatography flame ionization detector (GC‐FID) produced in (A) T175 flasks, i.e., 2D, (B) on plant‐based scaffolds, and (C) in 2D in culture flasks in the presence of plant‐based scaffolds. AA indicates ascorbic acid.

When exploring the effect of different media formulations on the lipid profile of 2D cultivated fat, no significant difference in fatty acid compositions was found between samples cultivated with and without intralipid in α‐MEM‐based supplemented with 1% NouS media (Figure 5A). However, when intralipid was added to FBS‐containing α‐MEM and DMEM/F12 supplemented with ascorbic acid significant changes were observed. Oleic acid (C18:1), linoleic acid (C18:2), and α‐linolenic acid (C18:3) shares decreased, while that of stearic acid (C18:0) increased compared to intralipid‐free conditions (Figure 7A).

A similar trend was observed in 3D‐cultivated samples. The addition of intralipid to FBS‐containing media leads to increased amounts of stearic acid (C18:0) and decreased amounts of oleic (C18:1), linoleic (C18:2), and alpha‐linolenic (C18:3) acids. Minor variations were noted between samples cultured in FBS‐containing α‐MEM and those in FBS‐containing DMEM/F12 supplemented with ascorbic acid, with the latter exhibiting slightly elevated levels of arachidic (C20:0) and behenic (C22:0) fatty acids. The choice of scaffold material did not significantly impact the lipid profile of the 3D‐cultivated fat, with the exception of samples cultured in FBS‐containing α‐MEM without intralipid. In this specific condition, potato‐soy scaffolds demonstrated higher concentrations of stearic acid (C18:0) and lower concentrations of oleic (C18:1) and linoleic (C18:2) acids compared to those cultivated on potato‐based scaffolds. Due to inadequate cell viability observed in α‐MEM NouS‐containing with intralipid on potato scaffolds, this condition was subsequently excluded from further analysis (Figure 7B). When the lipid profile of fat cultivated in 2D in the presence of a scaffold was analyzed, the previously observed findings were confirmed (Figure 7C). However, the observed differences in fatty acid profiles highlight the significant impact of changes in the feeding media given by the particles from the scaffolds. This result does not completely underline the observed fatty acid ratios in the literature. Yuen et al. described intralipid‐supplemented murine adipogenic precursor cells and found an increase in palmitic (C16:0) content and a decrease of stearic acid (C18:0) content [63]. This contrasts with the results of our analysis, which showed that stearic content increased in pAD‐MSC supplemented with intralipid. In consideration of the general media composition, α‐MEM and DMEM/F12 with ascorbic acid were compared and showed only minor differences.

Regarding the lipid profile analysis, the potential confounding effect of exogenous intralipid on the lipid composition analysis was addressed. All differentiated cells and cell‐laden scaffolds were thoroughly washed at least three times with PBS prior to lipid extraction. This washing protocol is widely used to remove residual media components and is considered safe for maintaining cell integrity. While this approach substantially reduces the presence of extracellular intralipid, it cannot be completely ruled out that not all traces of exogenous lipids were removed, and therefore, a minor fraction of the detected lipids may originate from residual intralipid rather than being synthesized by the cells.

## 4 Conclusion

The findings of the current study demonstrate that the biocompatibility and cell adhesion of plant protein‐based scaffolds, can be improved by coating them with vitronectin and poly‐L‐lysine, especially low molecular weight εPLL, the latter being a more cost‐effective solution. In contrast, atmospheric cold plasma treatment does not appear to improve cell adhesion and proliferation on these scaffolds; however, it is possible that oxygen or nitrogen cold plasma treatment may have more significant effects.  Investigating the impact of the use of different gases in cold plasma on scaffold modification could provide valuable insights for optimization of the biological performance of these materials, and should be considered as an important direction for future research.

For the adipogenic differentiation of pAD‐MSC on scaffolds, DMEM/F12 media supplemented with ascorbic acid, FBS, and intralipid was identified as the most suitable formulation, particularly when utilizing potato‐based scaffolds as the matrix. Furthermore, while the choice of scaffold material and the composition of the basal media had a minimal impact on the lipid profile, the inclusion of intralipid resulted in an increase in the proportion of stearic acid and a decrease in the proportions of oleic, linoleic, and alpha‐linolenic acids. These results demonstrate that modifying the culture media formulation, specifically through the addition of fatty acid emulsions (intralipid), enables the alteration of the lipid profile in cultured fat. Notably, the fatty acids distribution in all samples cultivated with intralipid demonstrates the increased share of stearic acid, which has a low (1.4%–5.5%) share in soybean oil—the lipid component of intralipid [76]. This could be evidence that fatty acids are not simply absorbed by the cells or scaffolds, but are actively taken up and metabolized, leading to the reduction of oleic acid to stearic acid or even de novo synthesis in differentiated pAD‐MSCs. To distinguish between these two possibilities, established methodologies such as stable isotope tracing with ^13^C‐labeled fatty acids or other labeled substrates can be employed. By supplementing the culture with ^13^C‐labeled fatty acids and subsequently analyzing the incorporation of the label into cellular lipids using mass spectrometry or NMR, it is possible to differentiate between exogenously incorporated and newly synthesized fatty acids. However, as these analyses would have exceeded the scope of the study, no tracer‐based metabolic analysis was performed in this study.

The results of this work highlight the potential of optimizing scaffold coatings and media formulations to improve the viability and functional properties of cultivated meat products and can contribute to future advances in creating tailored, sustainable, and nutritionally balanced alternatives to conventional meat.

## Author Contributions

**Mariya Abyzova**: conceptualization, development of methodology, experiment performance and data analysis, writing – original draft preparation, writing – review and editing. **Lasse Schoppe**: development of methodology, experiment performance, and data analysis. **Marline Kirsch**: writing – review and editing, data analysis. **Martin Muuß**: fatty acid composition measurements, data analysis. **Sina Zargarchi**: cold plasma treatment of scaffolds, data analysis. **Jordi Morales‐Dalmau**: writing – review and editing, data analysis. **Tuba Esatbeyoglu**: writing – review and editing, data analysis. **Ulrich Krings**: writing – review and editing, data analysis of fatty acid composition. **Antonina Lavrentieva**: conceptualization, writing – review and editing, supervision, funding acquisition.

## Conflicts of Interest

Mariia Abyzova, Marline Kirsch, and Jordi Morales‐Dalmau are employees of Cultimate Foods GmbH (Berlin/Hannover). However, the authors declare that they wrote this manuscript objectively and without bias. The company did not influence manuscript preparation. No other conflicts of interest exist.

## Data Availability

The raw data supporting the findings of this study are available from the authors upon reasonable request.
